# Potential misinformation in websites on carpal tunnel syndrome

**DOI:** 10.1016/j.pecinn.2024.100323

**Published:** 2024-07-15

**Authors:** Ria Goyal, Grace Corrier, David Ring, Amirreza Fatehi, Sina Ramtin

**Affiliations:** Department of Surgery and Perioperative Care Dell Medical School – The University of Texas at Austin, 1701 Trinity Street, Austin, TX 78712, USA

**Keywords:** Carpal tunnel syndrome, Median nerve, Misinformation, Website

## Abstract

**Objective:**

We sought to evaluate the potential reinforcement of misconceptions in websites discussing carpal tunnel syndrome (CTS).

**Methods:**

After removing all cookies to limit personalization, we entered “carpal tunnel syndrome” into five search engines and collected the first 50 results displayed for each search. For each of the 105 unique websites, we recorded publication date, author background, and number of views. The prevalence of potential reinforcement and/or reorientation of misconceptions for each website was then scored using a rubric based on our interpretation of the best current evidence regarding CTS. The informational quality of websites was graded with the DISCERN instrument, a validated tool for assessing online health information.

**Results:**

Every website contained at least one potentially misleading statement in our opinion. The most common misconceptions reference “excessive motion” and “inflammation.” Greater potential reinforcement of misinformation about CTS was associated with fewer page views and lower informational quality scores.

**Conclusions:**

Keeping in mind that this analysis is based on our interpretation of current best evidence, potential misinformation on websites addressing CTS is common and has the potential to increase symptom intensity and magnitude of incapability via reinforcement of unhelpful thoughts regarding symptoms.

**Innovation:**

The prevalence of patient-directed health information that can increase discomfort and incapability by reinforcing common unhelpful thoughts supports the need for innovations in how we develop, oversee, and evolve healthy online material.

## Highlights

• Potential misinformation on websites addressing CTS is common.
• Misinformation on websites has the potential to increase symptom intensity.
• Greater reinforcement of misinformation was associated with fewer page views.

## 1 Introduction

The internet is a widely used source of health information, with >70% of web users indicating that they have searched for health-related information online [1]. However, the quality of health information online varies [2,3]. For example, in the musculoskeletal realm, an investigation of websites discussing carpal tunnel syndrome (CTS) found that they were fairly difficult for most people to read and understand, with very few being at or below the recommended 6th-grade reading level [4]. A study of the informational quality of websites that address CTS concluded that most were written by authors with insufficient credentials and had low informational value [5]. An updated investigation by the same authors a decade later found that there was modest improvement in reliability and quality [6].

These studies mainly focused on the completeness of CTS diagnosis and treatment information provided, but they also noted the presence of unconventional and misleading information, particularly in association with financial motivations. We believe that the definition of misleading can be broadened to include notions that could reinforce misconceptions, or thoughts about symptoms that can negatively influence patient behavior and outcomes. Both symptom intensity and magnitude of incapability are greater in association with more unhelpful thoughts such as worst-case thinking [7,8]. Online health information that might reinforce unhelpful thoughts or feelings of distress has the potential for physical/iatrogenic, psychological, and financial harm. For instance, a suggestion that activity causes CTS can reinforce fear of movement (kinesiophobia) or worst-case thinking, both of which are specific examples of unhelpful thinking that have notable associations with greater pain intensity and greater magnitude of incapability [9,10]. Examining the prevalence of website information with the potential to reinforce common unhelpful thoughts about symptoms (misconceptions) might help increase physician awareness of the amount of unhealthy information people are exposed to online, enabling them to anticipate these views and reorient misconceptions both in clinic and online [11].

We sought to understand the prevalence and nature of information on websites regarding CTS that has the potential to reinforce common misconceptions and unhelpful thinking about symptoms. 1) Are there factors associated with the potential reinforcement and reorientation of misconception scores amongst websites covering carpal tunnel syndrome (CTS)? 2) Does a correlation exist between these scores and information quality scores?

## 2 Methods

Institutional Review Board (IRB) approval was not required for this study, as it involved analysis of publicly available information. In July 2020, we entered “carpal tunnel syndrome” into five of the most commonly used search engines: Google, Bing, Yahoo, Ask, and Duckduckgo. We intended our search strategy to mimic what the average person might encounter. All internet cookies were disabled to mitigate personalization bias. The first 50 websites displayed were collected, and the frequency that each article appeared on each unique search engine was noted so as to prevent duplicate scoring. Initial website queries and statistical data acquisition were completed in the same day to ensure the most relevant data was obtained and was representative of the same time period. After excluding duplicates and websites not focused on carpal tunnel syndrome, 105 unique websites were assessed.

For each article, we recorded the search engine where it was ranked the highest (and thus most likely to be accessed by a patient), the search rank by relevance, the publication date, author background, sponsorship status, and the number of views. The number of views the article received was determined using the program “Alexa Rank.” This is a global ranking system that determines the popularity of a website relative to other sites by analyzing the site's traffic and engagement of internet viewers over the past 3 months [12]. Additionally, if the article author was a medical professional with credentials that were easily located on the page, the author was recorded as qualified. If the author could not be identified or was not a medical professional, the author was recorded as unqualified.

The primary outcome of our study was the presence of potentially misleading information presented in websites regarding carpal tunnel syndrome. We used a scoring system previously designed to grade websites for the inclusion of potential misinformation that reinforced notions that, in our opinion, are contrary to best current evidence and expert opinion, or represent areas of debate, and have the potential to reinforce unhelpful thinking associated with greater levels of discomfort and incapability [11]. In other words, we measured ideas that are potentially harmful to one's health. A point was deducted for each statement on the website met one of the criteria for potential reinforcement of misconceptions. A point was added for each accurate statement with potential to reorient common misconceptions about idiopathic median neuropathy at the carpal tunnel. The resulting Reinforcement and Reorientation of Potential Misconception scores ranged from −12 to 12; with lower scores indicating a greater amount of potentially misleading content.

Information quality was determined with the DISCERN scoring handbook. The DISCERN Instrument is a validated tool used to grade the quality of written health information and includes an assessment of information source reliability. We reported Information Quality scores on a scale of 0 to 15, where 15 suggested the dissemination of the highest quality of information.

Two reviewers independently calculated Potential Misinformation and Information Quality scores on a subset of websites (*n* = 30) to evaluate for interobserver reliability of the grading tool using Intraclass Correlation Coefficient (ICC). After confirming an ICC of 0.71(moderate) [13], a single reviewer independently assessed Potential Misinformation and Information Quality scores for the videos.

We performed descriptive statistics on website characteristics (Table 1). We calculated Spearman rank correlation coefficients (ρ) to evaluate associations between non-normally distributed variables, Potential Misinformation scores, and Information Quality scores. We used a Kruskal-Wallis Test to assess differences in Potential Misinformation scores and Information Quality scores between websites. *P* values below 0.05 were considered significant. In bivariate analysis of Potential Misinformation scores and Information Quality scores, we used Poisson regression model. For variables with a *P* value below 0.05 demonstrating collinearity on variance inflation factor analysis, we included only the single variable amongst these that exhibited best fit in Poisson model. We then took variables that were significant in this analysis and moved them to a linear regression model.

**Table 1:** Descriptive Analysis of Websites.

	Median (IQR)
**Discern Score**	32 (23–38)
**Days since publication**	521 (282–1085)
Unknown	41% (43)
**Alexa rank score**	30,231 (3465–192,807)
N/A	2% (2)

	Mean ± SD (Range)
**Potential Reinforcement and Reorientation of Misconceptions**	2.2 ± 3.5 (−7 to 10)

Search Engines	Percentage of websites (Number)
Google	39% (41)
Ask	28% (29)
Bing	25% (26)
DDG	4% (5)
Yahoo	3% (4)
**Authors background**	
Not qualified/could not verify the authors credentials	65% (68)
Author is a qualified medical professional	35% (37)
**Sponsored/Biased**	31% (33)
**Article content**	
Conventional (explains diagnosis, symptoms, and treatment accurately)	59% (62)
Nonconventional (provides accurate information but in a non-traditional format - ex. explaining CTS only in pregnant women)	30% (32)
Misleading (provides inaccurate information)	2% (2)
Noninformational (is a website that is designed more like an advertisement and doesn't truly explain CTS)	8% (9)

## 3 Results

### 3.1 Prevalence of potential reinforcement of misconceptions

3.1

Every website contained at least one statement with the potential to reinforce misconceptions about carpal tunnel syndrome. The most prevalent potential misconceptions (unhelpful thoughts) were the notions that CTS is an inflammatory process (in 70% of websites; 73 of 105), and that CTS is caused by activity (“repetitive motion” or excessive hand use; 46% of websites; 48 of 105) (Table 2). The most-common correct statements about CTS regarded the pathophysiology as compression of the median nerve in the carpal tunnel (in 98% of websites; 103 of 105) and that the primary symptom is paresthesia (67% of websites; 71 of 105). The mean potential reinforcement and reorientation score was 2.2 points, indicating relative balance with a slight tendency towards avoidance of potential misinformation, with a standard deviation of 3.5 points, indicating notable variability. Nearly a third of websites were either sponsored by a commercial product or promoted one (31%; 33 of 105). Nearly two-thirds of websites had unqualified authors, or authors with unverifiable credentials (65%; 68 of 105) (Table 1).

**Table 2:** Distribution of Potential Misconceptions and Reorientations.

Inclusion of Potential Misconceptions	
Pain is more prominent than numbness	31% (33)
Numbness is located in the entire hand (including the small finger)	25% (26)
CTS is an inflammatory process	70% (73)
CTS is caused by repetitive motion (e.g. typing)	46% (48)
Bracing is disease-modifying (curative)	37% (39)
Exercises are disease-modifying	15% (16)
Traditional/alternative treatments are disease-modifying	13% (14)
Corticosteroids are disease-modifying	22% (23)
NSAIDs are disease-modifying	14% (15)
X-Ray imaging is a component of treatment	19% (21)
Surgical release is often unsuccessful	2% (2)
Surgical treatment requires prolonged recovery	3% (3)

Inclusion of Potential Reorientation of Misconceptions	
Pathophysiology involves median nerve compression in the carpal canal	98% (103)
The main symptom is numbness/tingling	67% (71)
CTS is idiopathic (Has an unknown cause)	30% (31)
CTS has a genetic basis	33% (35)
CTS typically occurs only in adults	36% (38)
Symptoms occur primarily at night	64% (67)
Surgery is the only known disease-modifying treatment	39% (41)
Steroid injections are temporary at best	30% (31)
CTS is eventually bilateral	13% (14)
Neuropathology advances with age	30% (31)
CTS can lead to permanent muscle atrophy	48% (50)
Reoccurrence after surgery is rare	35% (37)

### 3.2 Factors associated with potential reinforcement and reorientation of misconception scores

In bivariate analysis, higher potential reinforcement and reorientation of misconception scores (indicating the inclusion of fewer misleading notions) were independently associated with better information quality (higher modified DISCERN scores; correlation coefficient = 0.55, *p* ≤0.001) and a lower Alexa rank score (correlation coefficient = −0.25, *p* = 0.01) (Table 3). This association between the potential misconception score and information quality score persisted in linear regression analysis (regression coefficient = 0.2; 95% CI = 0.14–0.25; partial r-square = 0.31; *p* = 0.001) (Table 4).

**Table 3:** Bivariate Analysis of Factors Associated with Potential Reinforcement and Reorientation of Misconceptions.

Independent Variable	Rho	*P*-value
Discern Score	0.55	**<0.001**
Days since publication	−0.13	0.32
Alexa rank score	−0.25	**0.01**

Search Engines	**Mean (SD)**	0.38
Google	1.9 (3.1)	
Ask	2.3 (3.3)	
Bing	2.1 (4.1)	
DDG	3.2 (4.9)	
Yahoo	3.5 (4.7)	

Authors background		0.37
Not qualified/could not verify the authors credentials	2.0 (3.6)	
Author is a qualified medical professional	2.6 (3.4)	

Sponsored/Biased		0.86
Yes	2.1 (3.6)	
No	2.3 (3.5)

**Table 4:** Linear Regression Model for Factors Associated with Potential Reinforcement and Reorientation of Misconceptions.

Independent Variable	Regression Coefficient (CI)	Standard Error	P-value
Discern Score	0.2 (0.14 to 0.25)	0.03	**0.001**
Alexa rank score	−0.0000008 (−0.000001 to 0.00000005)	0.0000004	0.07

## 4 Discussion and conclusion

### 4.1 Discussion

Given the prevalence of health information on the Internet, it is important to account for the potential for online health information to negatively impact health by reinforcing unhelpful thoughts and misconceptions associated with greater symptom intensity. We studied websites about CTS and identified several common misleading notions that could lead to reinforcement of unhealthy beliefs and behaviors. A greater prevalence of potential reinforcement of misconceptions was associated with lower website quality scores.

This study has several limitations. First, the frequency of newly published articles and the constantly changing algorithms of the different search engines used mean that, most likely, search ranks will shift over time. Nevertheless, we believe that the primary finding of prevalent misleading statements in these articles would apply just as well to a repeat search. Additionally, this study only includes information regarding idiopathic CTS that was shared on websites written in English. However, the presence of inaccurate or misleading online health information has been studied internationally as well, demonstrating that its prevalence and possible adverse effects to patients is not limited to those who speak English [[14], [15], [16]]. Finally, a level of subjectivity is inherent in our reinforcement rubric. The principles used included a focus on objective pathophysiology and impairment, experimental evidence, and avoidance of concepts that are debatable if they have the potential to reinforce unhelpful thinking. For instance, we interpret best evidence to indicate a natural history of progression of neuropathy and the only experimentally verified disease-modifying treatment (treatment that can limit neuropathy) is surgery. In the review process there was specifically some debate regarding the issue of whether surgery is the only proved disease-modifying treatment. One reviewer cited a 2011 cohort study of changes on ultrasound and electrodiagnostic parameters after corticosteroid injection [17]. A recent network metanalysis of randomized trials found modest transient improvement in nerve conduction after corticosteroid injection that were resolved by six months, pointing to a mostly palliative, and minimally and temporarily disease-modifying action [18]. We acknowledge that new evidence might alter our collective understanding of idiopathic median neuropathy at the carpal tunnel. In particular, the natural history of idiopathic median neuropathy at the carpal tunnel (course of the neuropathy untreated) and the ability of treatments to alleviate symptoms (palliative) or prevent or meaningfully delay the progression of the neuropathy (disease-modification). Such evidence would lead to changes in the potential misinformation checklist consistent with our guiding principles. This useful discussion highlights some of the potential sources of some areas of potential misinformation, including alternative interpretations of evidence, or persisting beliefs based on outdated or lower quality evidence. It evens calls out the possibility that our study might introduce misinformation if we are misinterpreting or over interpreting the best available evidence. There would likely be consensus that if there is an area of debate, it should be highlighted as such and the reasons for ongoing debate enumerated. We included this specific point regarding disease-modifying treatment as a potential source of misinformation because a person whose symptoms are alleviate might have the misunderstanding that the disease is cured, which could place them at risk of presenting years later with irreversible advanced neuropathy. The checklist as currently structured has acceptable interobserver reliability [11].

The observation that every website studied presented at least one statement with the potential to reinforce common unhelpful thoughts about CTS confirms the potential for online information to harm health. The inaccurate idea that the pathophysiology of carpal tunnel syndrome is inflammation, and the unsupported concept that it is caused by hand use—amongst other common misconceptions about CTS—have the potential to reinforce unhelpful thinking known to be associated with greater pain intensity and magnitude of incapability, catastrophic thinking and kinesiophobia in particular. While the mean score indicated a balance towards reorientation of unhelpful thinking, it was close to neutral and had a large standard deviation. These results suggest that people might benefit from access to a website scoring rubric of potential misinformation, especially when coupled with evidence that websites are often incomplete and biased in their presentation of CTS information [5,6]. Website developers can start from the principle that words and concepts influence health and strive to provide the healthiest possible explanation of a condition.

The observation that less potential reinforcement of misconceptions was independently associated with better information quality is consistent with a prior study on misleading information about CTS in YouTube videos, which found similar associations [11]. Another similarity is that there are no metrics available to the patient, such as search rank and author credentials, that associate with the presence of potentially misleading information. These observations support the idea that people cannot use search ranks or other data to identify the most helpful and healthful information provided by online sources.

### 4.2 Innovation

Evaluation and rating of websites for reading level, accuracy, and adequate support and citations are intended to improve the quality and utility of web-based material. They can be used by developers to improve quality and consumer to judge quality. Our innovation is to add another level of evaluation: identification of possible misinformation due to reinforcement of commonplace misconceptions and misinterpretation of symptoms that result from human mental heuristics and cognitive bias. Examples of these sorts of unhelpful thinking include worst-case thinking, interpretation of painful movement as harmful, and other unhealthy interpretations of sensations. Websites have the potential for harm and for benefit to the degree that they are aware of potential misinformation and take steps to limit or correct it.

### 4.3 Conclusion

Using an example of carpal tunnel syndrome, we demonstrated that online health information often makes statements with the potential reinforce unhelpful thoughts associated with greater discomfort and incapability. In other words, people seeking information and wellbeing, might experience greater levels of discomfort and incapability after reading online health information. Clinicians can anticipate such common unhelpful thoughts, understand that they have likely been reinforced by information on websites, and work to develop more accurate and healthy online material.

## Source of funding

None.

## CRediT authorship contribution statement

**Ria Goyal:** Writing – original draft, Methodology, Data curation. **Grace Corrier:** Writing – review & editing, Writing – original draft, Formal analysis. **David Ring:** Writing – review & editing, Writing – original draft, Validation, Supervision, Methodology, Investigation, Conceptualization. **Amirreza Fatehi:** Writing – review & editing, Supervision, Formal analysis, Data curation. **Sina Ramtin:** Writing – review & editing, Supervision, Methodology, Conceptualization.

## Declaration of competing interest

The authors declare the following financial interests/personal relationships which may be considered as potential competing interests: David Ring reports a relationship with Skeletal Dynamics LLC that includes: consulting or advisory. David Ring reports a relationship with Wolters Kluwer Health Inc. that includes: consulting or advisory. David Ring reports a relationship with Lawyers that includes: paid expert testimony. Deputy Editor for Clinical Orthopedics and Related Research (Dr. David Ring) If there are other authors, they declare that they have no known competing financial interests or personal relationships that could have appeared to influence the work reported in this paper.

## Appendix A Supplementary data

Appendix A: Websites evaluated (N = 105). Image 1
