# Supplementary material for: Potential misinformation in websites on carpal tunnel syndrome
Source: PEC Innov. 2024 Jul 15;5:100323. doi: 10.1016/j.pecinn.2024.100323 (PMC11325073; doi:10.1016/j.pecinn.2024.100323)
Supplement: Supplementary file 1 — Appendix A: Websites evaluated (N = 105). [file mmc1.docx]

**Appendix**. List of evaluated websites (N = 105).

1. 5 Ways to Ease Your Carpal Tunnel Symptoms. *Prevention.* (2015). [Accessed 2020]. <https://www.prevention.com/health/a20480815/prevent-carpal-tunnel-syndrome/>.
2. 10 Carpal Tunnel Symptoms. *10FAQHealth*. [Accessed 2020]. <https://www.10faq.com/health/carpal-tunnel-symptoms/>.
3. 10 Carpal Tunnel Syndrome Symptoms. *New Life Ticket*. [Accessed 2020]. <https://www.newlifeticket.com/conditions/10-carpal-tunnel-syndrome-symptoms/>.
4. 10 Symptoms of Carpal Tunnel Syndrome. *Very Healthy Life*. (2017). [Accessed 2020]. <https://veryhealthy.life/10-symptoms-carpal-tunnel-syndrome>.
5. 14 Symptoms of Carpal Tunnel. *Simply Health Today*. [Accessed 2020]. <http://simplyhealth.today/10-symptoms-carpal-tunnel/>.
6. Carpal Tunnel. *Corewell Health.* [Accessed 2020]. <https://www.beaumont.org/conditions/carpal-tunnel>.
7. Carpal Tunnel. Is it Causing Your Shoulder Pain? *Back to Motion Physical Therapy*. [Accessed 2020]. <https://backtomotion.net/carpal-tunnel/>.
8. Carpal Tunnel Syndrome. *Amboss*. (2020). Updated 2023. [Accessed 2020]. <https://www.amboss.com/us/knowledge/Carpal_tunnel_syndrome>.
9. Carpal Tunnel Syndrome. *American* *Academy* *of* *Family* *Physicians*. Updated 2020. [Accessed 2020]. <https://familydoctor.org/condition/carpal-tunnel-syndrome/>.
10. Carpal Tunnel Syndrome. *American* *Association* *of* *Neuromuscular* *&* *Electrodiagnostic* *Medicine.* [Accessed 2020]. <https://www.aanem.org/clinical-practice-resources/patient-information/muscle-nerve-disorders/disorder/carpal-tunnel-syndrome>.
11. Carpal Tunnel Syndrome. *American Society for Surgery of the Hand*. Updated 2021. [Accessed 2020]. <https://www.assh.org/handcare/condition/carpal-tunnel-syndrome>.
12. Carpal Tunnel Syndrome. *Arthritis Foundation*. [Accessed 2020]. <https://www.arthritis.org/diseases/carpal-tunnel-syndrome>.
13. Carpal Tunnel Syndrome. *Better Health, Victoria*. [Accessed 2020]. <https://www.betterhealth.vic.gov.au/health/conditionsandtreatments/carpal-tunnel-syndrome>.
14. Carpal Tunnel Syndrome. *Canadian Centre for Occupational Health and Safety*. Updated 2024. [Accessed 2020]. <https://www.ccohs.ca/oshanswers/diseases/carpal.html>.
15. Carpal Tunnel Syndrome. *Carle Health*. [Accessed 2020]. <https://carle.org/conditions/neurological-conditions/carpal-tunnel-syndrome>.
16. Carpal Tunnel Syndrome. *Cedars-Sinai*. [Accessed 2020]. <https://www.cedars-sinai.org/health-library/diseases-and-conditions/c/carpal-tunnel-syndrome.html>.
17. Carpal Tunnel Syndrome. *Cleveland Clinic.* [Accessed 2020]. <https://my.clevelandclinic.org/health/diseases/4005-carpal-tunnel-syndrome>.
18. Carpal Tunnel Syndrome. *Corewell Health*. [Accessed 2020]. <https://www.spectrumhealth.org/services/orthopedics/hand-microsurgery-and-upper-extremity/carpal-tunnel-syndrome>.
19. Carpal Tunnel Syndrome. *Drugs.com*. Updated 2023. [Accessed 2020]. <https://www.drugs.com/health-guide/carpal-tunnel-syndrome.html>.
20. Carpal Tunnel Syndrome. *DukeHealth*. [Accessed 2020]. <https://www.dukehealth.org/treatments/orthopaedics/carpal-tunnel-syndrome>.
21. Carpal Tunnel Syndrome. *FitzHand,* [Accessed 2020]. <https://fitzhand.com/carpal-tunnel-syndrome/>.
22. Carpal Tunnel Syndrome. *The Foundation for Peripheral Neuropathy.* (2016). [Accessed 2020]. <https://web.archive.org/web/20200608230236/https://www.foundationforpn.org/what-is-peripheral-neuropathy/causes/carpal-tunnel-syndrome/>.
23. Carpal Tunnel Syndrome. *Healthline*. Updated 2019. [Accessed 2020]. <https://www.healthline.com/health/carpal-tunnel-syndrome>.
24. Carpal Tunnel Syndrome. *Healthwise, Kaiser Permanente.* Updated 2023. [Accessed 2020]. <https://healthy.kaiserpermanente.org/health-wellness/health-encyclopedia/he.carpal-tunnel-syndrome.hw213308>.
25. Carpal Tunnel Syndrome. *Johns Hopkins Medicine*. [Accessed 2020]. <https://www.hopkinsmedicine.org/health/conditions-and-diseases/carpal-tunnel-syndrome>.
26. Carpal Tunnel Syndrome. *M Health Fairview*. [Accessed 2020]. <https://www.mhealthfairview.org/conditions/carpal-tunnel-syndrome>.
27. Carpal Tunnel Syndrome. *Mayo* *Clinic*. Updated 2024. [Accessed 2020]. <https://www.mayoclinic.org/diseases-conditions/carpal-tunnel-syndrome/symptoms-causes/syc-20355603>.
28. Carpal Tunnel Syndrome. *MedlinePlus*. [Accessed 2020]. <https://medlineplus.gov/genetics/condition/carpal-tunnel-syndrome/>.
29. Carpal Tunnel Syndrome. *MedStar Health.* [Accessed 2020]. <https://www.medstarhealth.org/services/carpal-tunnel-syndrome>.
30. Carpal Tunnel Syndrome. *Memorial* *Sloan* *Kettering* *Cancer* *Center.* [Accessed 2020]. <https://www.mskcc.org/cancer-care/patient-education/carpal-tunnel-syndrome>.
31. Carpal Tunnel Syndrome. *Mount Sinai*. [Accessed 2020]. <https://www.mountsinai.org/health-library/diseases-conditions/carpal-tunnel-syndrome>.
32. Carpal Tunnel Syndrome. *National Health System*. [Accessed 2020]. <https://www.nhs.uk/conditions/Carpal-tunnel-syndrome/>.
33. Carpal Tunnel Syndrome. *Nemours KidsHealth.* [Accessed 2020]. <https://kidshealth.org/en/kids/carpal.html/>.
34. Carpal Tunnel Syndrome. *Office on Women’s Health*. Updated 2021 [Accessed 2020]. <https://www.womenshealth.gov/a-z-topics/carpal-tunnel-syndrome>.
35. Carpal Tunnel Syndrome. *Oregon Health & Science University Brain Institute.* [Accessed 2020]. <https://www.ohsu.edu/brain-institute/carpal-tunnel-syndrome>.
36. Carpal Tunnel Syndrome. *Orthobullets*. Updated 2024. [Accessed 2020]. <https://www.orthobullets.com/hand/6018/carpal-tunnel-syndrome>.
37. Carpal Tunnel Syndrome. *Orthogate*. (2006). [Accessed 2020]. <https://web.archive.org/web/20210922194013/https://www.orthogate.org/patient-education/hand/carpal-tunnel-syndrome>.
38. Carpal Tunnel Syndrome. *OrthoInfo*, *American Academy of Orthopaedic Surgeons*. Updated 2022. [Accessed 2020]. <https://orthoinfo.aaos.org/en/diseases--conditions/carpal-tunnel-syndrome/>.
39. Carpal Tunnel Syndrome. *Penn Medicine*. [Accessed 2020]. <https://www.pennmedicine.org/for-patients-and-visitors/patient-information/conditions-treated-a-to-z/carpal-tunnel-syndrome>.
40. Carpal Tunnel Syndrome. *Physiopedia*. [Accessed 2020]. <https://www.physio-pedia.com/Carpal_Tunnel_Syndrome>.
41. Carpal Tunnel Syndrome. *Southern California Orthopedic Institute*. [Accessed 2020]. <https://www.scoi.com/patient-resources/education/carpal-tunnel-syndrome>.
42. Carpal Tunnel Syndrome. *Stanford Health Care*. [Accessed 2020]. <https://stanfordhealthcare.org/medical-conditions/bones-joints-and-muscles/carpal-tunnel-syndrome/treatments.html>.
43. Carpal Tunnel Syndrome. *Summit Orthopedics*. [Accessed 2020]. <https://www.summitortho.com/find-care/services-conditions/hand-wrist/hand-wrist-related-conditions-treatments/carpal-tunnel-syndrome/>.
44. Carpal Tunnel Syndrome. *Teach Me Surgery*. Updated 2022. [Accessed 2020]. <https://teachmesurgery.com/orthopaedic/wrist-and-hand/carpal-tunnel-syndrome/>.
45. Carpal Tunnel Syndrome. *University of Arkansas for Medical Sciences.* [Accessed 2020]. <https://uamshealth.com/condition/carpal-tunnel-syndrome/>.
46. Carpal Tunnel Syndrome. *UC San Diego Health.* Removed in 2021. [Accessed 2020]. <https://web.archive.org/web/20211017093402/https://health.ucsd.edu/specialties/surgery/ortho/hand/Pages/carpal-tunnel.aspx>.
47. Carpal Tunnel Syndrome. *University of Kentucky Health Care*. [Accessed 2020]. <https://ukhealthcare.uky.edu/orthopaedic-surgery-sports-medicine/conditions/general-orthopaedics/carpal-tunnel>.
48. Carpal Tunnel Syndrome. *University of Maryland Medical Center.* [Accessed 2020]. <https://www.umms.org/ummc/health-services/orthopedics/services/hand-wrist/carpal-tunnel-syndrome>.
49. Carpal Tunnel Syndrome. *University of Michigan Health*. [Accessed 2020]. <https://www.uofmhealth.org/conditions-treatments/hand-program/carpal-tunnel-syndrome>.
50. Carpal Tunnel Syndrome. *University of Rochester Medical Center*. [Accessed 2020]. <https://www.urmc.rochester.edu/encyclopedia/content.aspx?ContentTypeID=85&ContentID=P00048>.
51. Carpal Tunnel Syndrome. *University of Utah Health*. [Accessed 2020]. <https://healthcare.utah.edu/orthopaedics/specialties/hand-pain/carpal-tunnel>.
52. Carpal Tunnel Syndrome. *UW Medicine.* [Accessed 2020]. <https://orthop.washington.edu/patient-care/articles/arthritis/carpal-tunnel-syndrome.html>.
53. Carpal Tunnel Syndrome. *Very Well Health*. Updated 2023. [Accessed 2020]. <https://www.verywellhealth.com/carpal-tunnel-syndrome-4012826>.
54. Carpal Tunnel Syndrome. *Virginia Mason Franciscan Health.* [Accessed 2020]. <https://web.archive.org/web/20210226222308/https://www.virginiamason.org/carpal-tunnel>.
55. Carpal Tunnel Syndrome. *WebMD*. Updated 2024. [Accessed 2020]. <https://www.webmd.com/pain-management/carpal-tunnel/carpal-tunnel-syndrome#1>.
56. Carpal Tunnel Syndrome. *Wikipedia*. Updated 2024. [Accessed 2020]. <https://en.wikipedia.org/wiki/Carpal_tunnel_syndrome>.
57. Carpal Tunnel Syndrome. *Yale Medicine*. [Accessed 2020]. <https://www.yalemedicine.org/conditions/carpal-tunnel-syndrome/>.
58. Carpal Tunnel Syndrome/Repair. *Lawrence Memorial Hospital Health*. [Accessed 2020]. <https://www.lmh.org/get-care/orthokansas/hand-wrist/carpal-tunnel-syndrome-repair/>.
59. Carpal Tunnel Syndrome Causing You Pain? *Jamestown Regional Medical Center*. (2020). [Accessed 2020]. <https://jrmcnd.com/hands-hurt-carpal-tunnel/>.
60. Carpal Tunnel Syndrome Information Page. *National Institutes of Health.* [Accessed 2020]. <https://web.archive.org/web/20200613214836/https://www.ninds.nih.gov/Disorders/All-Disorders/Carpal-Tunnel-Syndrome-Information-Page>.
61. Carpal Tunnel Syndrome Fact Sheet. *National Institutes of Health*. [Accessed 2020]. <https://web.archive.org/web/20200625231234/https://www.ninds.nih.gov/Disorders/Patient-Caregiver-Education/Fact-Sheets/Carpal-Tunnel-Syndrome-Fact-Sheet>.
62. Carpal Tunnel Syndrome: Tingling, Numbness and Pain. *Detroit Medical Center*. [Accessed 2020]. <https://www.dmc.org/services/orthopedics/orthopedic-healthy-living/corporate-content/carpal-tunnel-syndrome-tingling-numbness-and-pain>.
63. Carpal Tunnel Syndrome Treatment. *Providence Health & Services*. Removed in 2020. [Accessed 2020]. <https://web.archive.org/web/20200925211543/https://washington.providence.org/services-directory/services/c/carpal-tunnel-syndrome-treatment>.
64. Carpal Tunnel Treatment. *Robert Wood Johnson Barnabas Health.* [Accessed 2020]. <https://www.rwjbh.org/treatment-care/orthopedics/conditions-treatments/carpal-tunnel/>.
65. Don’t Delay Treatment for Carpal Tunnel Syndrome. *Harvard Health Publishing*. (2019). [Accessed 2020]. <https://www.health.harvard.edu/diseases-and-conditions/dont-delay-treatment-for-carpal-tunnel-syndrome>.
66. Everything You Need to Know About Carpal Tunnel Syndrome. *Beta Healthy*. [Accessed 2020]. <https://betahealthy.com/everything-you-need-to-know-about-carpal-tunnel-syndrome-symptomssignscauses-risk-factors-and-treatments/>.
67. How To Get Relief for Carpal Tunnel Pain at Home. *Cleveland Clinic*. Updated 2021. [Accessed 2020]. <https://health.clevelandclinic.org/ways-to-ease-carpal-tunnel-syndrome-pain-without-surgery>.
68. How to Treat Carpal Tunnel Syndrome. *WikiHow*. Updated 2024. [Accessed 2020]. <https://www.wikihow.health/Treat-Carpal-Tunnel-Syndrome>.
69. Methods Of Treating Carpal Tunnel Syndrome. *HealthPrep*. Updated 2023. [Accessed 2020]. <https://healthprep.com/joint-conditions/methods-treating-carpal-tunnel-syndrome/>.
70. Our Approach to Treating Carpal Tunnel Syndrome. *NYU Langone Health.* [Accessed 2020]. <https://nyulangone.org/conditions/carpal-tunnel-syndrome>.
71. Self Test for Carpal Tunnel Syndrome. *My Carpal Tunnel*. [Accessed 2020]. <https://www.mycarpaltunnel.com/self-test-carpal-tunnel/>.
72. Treatments for Carpal Tunnel. *Questions Answered*. Updated 2019. [Accessed 2020]. <https://www.questionsanswered.net/article/treatments-carpal-tunnel>.
73. Trigger Thumb and Carpal Tunnel Syndrome Connection. *Healthfully*. Removed 2021. [Accessed 2020]. <https://web.archive.org/web/20200921060635/https://healthfully.com/trigger-thumb-carpal-tunnel-syndrome-connection-7135.html>.
74. Try out These Exercises for Carpal Tunnel Syndrome and Say Goodbye to Nagging Pain. *HowtoCure.com*. Updated 2020. [Accessed 2020]. <https://howtocure.com/exercises-for-carpal-tunnel/>.
75. Understanding Carpal Tunnel Syndrome. *US Department of Veterans Affairs*. [Accessed 2020]. <https://web.archive.org/web/20211216212307/https://www.veteranshealthlibrary.va.gov/142,82403_VA>.
76. Understanding Carpal Tunnel Syndrome. *Virginia Spine Institute.* [Accessed 2020]. <https://www.spinemd.com/conditions/carpal-tunnel-syndrome/>.
77. What is Carpal Tunnel? *My Carpal Tunnel*. [Accessed 2020]. <https://www.mycarpaltunnel.com/carpal-tunnel-questions/what-is-carpal-tunnel/>.
78. What is Carpal Tunnel Syndrome? *Airrosti Rehab Center*. [Accessed 2020]. <https://www.airrosti.com/injuries-we-treat/carpal-tunnel-syndrome/>.
79. What is Carpal Tunnel Syndrome? *University of Pittsburgh Medical Center*. (2015). Updated 2022. [Accessed 2020]. <https://share.upmc.com/2015/12/what-is-carpal-tunnel-syndrome/>.
80. What Is Carpal Tunnel Syndrome? Your Guide. *Healthgrades.* Updated 2022. [Accessed 2020]. <https://www.healthgrades.com/right-care/carpal-tunnel-surgery/carpal-tunnel-syndrome>.
81. What triggers carpal tunnel? *The Centers for Advanced Orthopaedics.* (2020). [Accessed 2020]. <https://www.mdbonedocs.com/what-triggers-carpal-tunnel/>.
82. N. Ashworth. Carpal Tunnel Syndrome. *Medscape, WebMD*. Updated 2024. [Accessed 2020]. <https://emedicine.medscape.com/article/327330-overview?form=fpf>.
83. S. Bishop. What Can Happen to the Fingers if Carpal Tunnel Syndrome Is Left Untreated? *Mayo* *Clinic* *News* *Network*. (2012). [Accessed 2020]. <https://newsnetwork.mayoclinic.org/discussion/left-untreated-carpal-tunnel-syndrome-can-lead-to-weakness-in-fingers-and-thumb/>.
84. Y. Brazier. Carpal tunnel syndrome: What you need to know. *Healthline Media*. Updated 2023. [Accessed 2020]. <https://www.medicalnewstoday.com/articles/184337/>.
85. K. Chan. Carpal Tunnel Syndrome. *American College of Rheumatology*. Updated 2023. [Accessed 2020]. <https://rheumatology.org/patients/carpal-tunnel-syndrome>.
86. D. Christiano. What Causes Carpal Tunnel Syndrome During Pregnancy, and How’s It Treated? *Healthline Media*. (2018). [Accessed 2020]. <https://www.healthline.com/health/pregnancy/carpal-tunnel-pregnancy#outlook>.
87. W. Dubois. Carpal Tunnel Syndrome and Diabetes: What’s the Connection? *Healthline Media*. (2020). [Accessed 2020]. <https://web.archive.org/web/20200620171801/https://www.healthline.com/diabetesmine/carpal-tunnel-syndrome-and-diabetes#what-is-cts>.
88. N. Feuer. Carpal Tunnel Syndrome vs. Cervical Radiculopathy. *Veritas Health*. Updated 2017. [Accessed 2020]. <https://www.spine-health.com/conditions/neck-pain/carpal-tunnel-syndrome-vs-cervical-radiculopathy>.
89. K. Fultz. Carpal Tunnel Syndrome. *Blanchard Valley Health System.* [Accessed 2020]. <https://www.bvhealthsystem.org/expert-health-articles/carpal-tunnel-syndrome>.
90. N. Iftikhar. Carpal Tunnel vs. Arthritis: What’s the Difference? *Healthline Media*. (2020). [Accessed 2020]. <https://www.healthline.com/health/osteoarthritis/carpal-tunnel-vs-arthritis>.
91. K. E. Leblanc, W. Cestia. Carpal Tunnel Syndrome. *American* *Academy* *of* *Family* *Physicians*. (2011) [Accessed 2020]. <https://www.aafp.org/pubs/afp/issues/2011/0415/p952.html>.
92. A. Marcin. 9 Home Remedies for Carpal Tunnel Relief. *Healthline Media*. (2016). Updated 2020. [Accessed 2020]. <https://www.healthline.com/health/home-remedies-for-carpal-tunnel>.
93. D. Murray. Carpal Tunnel Syndrome in Pregnancy. *Very Well Family*. Updated 2021. [Accessed 2020]. <https://www.verywellfamily.com/carpal-tunnel-syndrome-in-pregnancy-4158977>.
94. H. Nichols. 10 Natural and Home Remedies for Carpal Tunnel Syndrome. *Healthline Media*. (2018). [Accessed 2020]. <https://www.medicalnewstoday.com/articles/314772>.
95. K. Repinski. Carpal Tunnel Syndrome vs. Arthritis: What’s the Difference? *CreakyJoints*. (2019). [Accessed 2020]. <https://creakyjoints.org/living-with-arthritis/symptoms/carpal-tunnel-vs-arthritis/>.
96. J. Schreiber. Carpal Tunnel Syndrome. *Joseph Schreiber, MD.* [Accessed 2020]. <https://www.schreibermd.com/carpal-tunnel>.
97. W. C. Shiel. Carpal Tunnel Syndrome. *EMedicineHealth*. [Accessed 2020]. <https://www.emedicinehealth.com/carpal_tunnel_syndrome/article_em.htm>.
98. W. C. Shiel. Carpal Tunnel Syndrome. *MedicineNet*. [Accessed 2020]. <https://www.medicinenet.com/carpal_tunnel_syndrome/article.htm>.
99. L. Shuer. Carpal Tunnel Syndrome. *American Association of Neurological Surgeons*. [Accessed 2020]. <https://www.aans.org/en/Patients/Neurosurgical-Conditions-and-Treatments/Carpal-Tunnel-Syndrome>.

100. C. Spader. Carpal Tunnel Release. *Healthgrades*. (2018). Updated 2020. [Accessed

2020]. <https://www.healthgrades.com/right-care/carpal-tunnel-surgery>.

101. D. Steinberg. Carpal Tunnel Syndrome (Consumer). *Merck Manual*. [Accessed 2020].

<https://www.merckmanuals.com/home/bone,-joint,-and-muscle-disorders/hand-disorders/carpal-tunnel-syndrome>.

102. D. Steinberg. Carpal Tunnel Syndrome (Professional). *Merck Manual*. [Accessed 2020].

<https://www.merckmanuals.com/professional/musculoskeletal-and-connective-tissue-disorders/hand-disorders/carpal-tunnel-syndrome>.

103. C. Tidy. Carpal Tunnel Syndrome. *Patient, Egton Medical Information Systems*. (2017).

Updated 2022. [Accessed 2020].

<https://patient.info/bones-joints-muscles/carpal-tunnel-syndrome-leaflet>.

104. L. Torborg. Mayo Clinic Q and A: Recovery after surgery for carpal tunnel syndrome —

what’s normal and what’s not. *Mayo Clinic News Network*. (2019). [Accessed 2020].

<https://newsnetwork.mayoclinic.org/discussion/mayo-clinic-q-and-a-recovery-after-surgery-for-carpal-tunnel-syndrome-whats-normal-and-whats-not/>.

105. J. M. Torpy. Carpal Tunnel Syndrome. *JAMA*. (2011). [Accessed 2020].

<https://jamanetwork.com/journals/jama/fullarticle/1104666>.
